# *Pseudomonas chlororaphis* PA23 metabolites protect against protozoan grazing by the predator *Acanthamoeba castellanii*

**DOI:** 10.7717/peerj.10756

**Published:** 2021-01-22

**Authors:** Akrm Ghergab, Carrie Selin, Jennifer Tanner, Ann Karen Brassinga, Teresa Dekievit

**Affiliations:** 1 Department of Microbiology, University of Manitoba, Winnipeg, Manitoba, Canada; 2 National Microbiology Laboratory, Public Health Agency of Canada, Winnipeg, Manitoba, Canada

**Keywords:** Pseudomonad, Amoebae, Predator-prey interaction, Biocontrol, Antibiotic, Gene expression, Trophozoite, Cyst

## Abstract

**Background:**

*Pseudomonas chlororaphis* strain PA23 is a biocontrol agent that is able to protect canola against the pathogenic fungus *Sclerotinia sclerotiorum*. This bacterium secretes a number of metabolites that contribute to fungal antagonism, including pyrrolnitrin (PRN), phenazine (PHZ), hydrogen cyanide (HCN) and degradative enzymes. In order to be successful, a biocontrol agent must be able to persist in the environment and avoid the threat of grazing predators. The focus of the current study was to investigate whether PA23 is able to resist grazing by the protozoan predator *Acanthamoeba castellanii* (Ac) and to define the role of bacterial metabolites in the PA23-Ac interaction.

**Methods:**

Ac was co-cultured with PA23 WT and a panel of derivative strains for a period of 15 days, and bacteria and amoebae were enumerated on days 1, 5, 10 and 15. Ac was subsequently incubated in the presence of purified PRN, PHZ, and KCN and viability was assessed at 24, 48 and 72 h. Chemotactic assays were conducted to assess whether PA23 compounds exhibit repellent or attractant properties towards Ac. Finally, PA23 grown in the presence and absence of amoebae was subject to phenotypic characterization and gene expression analyses.

**Results:**

PRN, PHZ and HCN were found to contribute to PA23 toxicity towards Ac trophozoites, either by killing or inducing cyst formation. This is the first report of PHZ-mediated toxicity towards amoebae. In chemotaxis assays, amoebae preferentially migrated towards regulatory mutants devoid of extracellular metabolite production as well as a PRN mutant, indicating this antibiotic has repellent properties. Co-culturing of bacteria with amoebae led to elevated expression of the PA23 *phzI*/*phzR* quorum-sensing (QS) genes and *phzA* and *prnA*, which are under QS control. PHZ and PRN levels were similarly increased in Ac co-cultures, suggesting that PA23 can respond to predator cues and upregulate expression of toxins accordingly.

**Conclusions:**

PA23 compounds including PRN, PHZ and HCN exhibited both toxic and repellent effects on Ac. Co-culturing of bacteria and amoebae lead to changes in bacterial gene expression and secondary metabolite production, suggesting that PA23 can sense the presence of these would-be predators and adjust its physiology in response.

## Introduction

*Pseudomonas chlororaphis* strain PA23 is a biocontrol agent capable of suppressing disease caused by the fungal pathogen *Sclerotinia sclerotiorum* (Fernando et al., 2007; Savchuk & Fernando, 2004). This bacterium produces an arsenal of secondary metabolites, which contribute to fungal antagonism. Secreted compounds include the diffusible antibiotics phenazine (PHZ) and pyrrolnitrin (PRN) together with hydrogen cyanide (HCN), protease, chitinase, and lipase (Poritsanos et al., 2006; Zhang et al., 2006). Mutants deficient in either PRN or HCN production exhibit reduced fungal inhibition, indicating that these two products are important for PA23 biocontrol (Selin et al., 2010; Nandi et al., 2017). While PHZ plays a more minor role in pathogen suppression, it does contribute to biofilm formation by this bacterium (Selin et al., 2010). A complex regulatory network that functions at the transcriptional and post-transcriptional level governs expression of these metabolites. For example the GacS-GacA two-component system, which works in concert with a second network called Rsm, acts as a positive regulator of PA23 biocontrol (Poritsanos et al., 2006; Selin et al., 2014). Similarly, the PhzRI quorum-sensing (QS) system activates expression of biocontrol genes; while RpoS and the sigma regulator PsrA function as repressors through downregulation of PRN biosynthetic genes (Manuel et al., 2012; Selin, Fernando & De Kievit, 2012; Selin et al., 2014).

Beyond its ability to suppress the disease-causing pathogen, the success of a biocontrol agent is contingent upon successful colonization of a given environment. One of the primary threats to environmental persistence is consumption by microfaunal predators, including protozoa and nematodes that feed upon bacteria. In response, bacteria have evolved strategies to help resist predation. One such antipredator defence tactic is the production of compounds with toxic and or repellent activities (Ekelund & Rõnn, 1994; Jousset, 2012; Philippot et al., 2013). We have previously demonstrated that PRN and HCN produced by PA23 exhibit nematocidal and repellent activities towards the nematode *Caenorhabditis elegans* (Nandi et al., 2015). Moreover, co-culturing leads to increased expression of genes and products associated with biocontrol, indicating that PA23 is able to sense and respond to the presence of *C. elegans* (Nandi et al., 2015).

In the soil, naked amoebae are key drivers of microbial community structure and activity due to their ability to access small pores (Ekelund & Rõnn, 1994). *Acanthamoeba castellani* (Ac) has been used as a model organism to explore bacteria-amoebae interactions. The life cycle of Ac is comprised of two stages: a vegetative trophozoite and a dormant cyst form. Trophozoites are covered with spindle-like surface projections known as acanthopodia, which are believed to facilitate prey capture, adhesion to surfaces, and cell motility. Under harsh conditions, trophozoites can differentiate into non-dividing, highly resistant cysts (Khan, 2006; Marciano-Cabral & Cabral, 2003).

To date, the fate of strain PA23 in the presence of the grazing predator Ac has yet to be explored. The focus of the current study was to ascertain whether PA23 is able to persist in the presence of this amoeba and to define the role of exoproducts in the predator–prey interaction. Our findings revealed that PRN, PHZ and HCN have detrimental effects on trophozoite viability and therefore help to protect against protozoan grazing in vitro. Co-culturing with amoebae led to enhanced expression of secondary metabolite genes and products, suggesting that PA23 is able to detect the presence of amoebae and adjust its physiology accordingly.

## Materials and Methods

### Bacterial strains and growth conditions

All bacterial strains and plasmids used in this study are listed in Table 1. *Escherichia coli* was cultured at 37 °C on Lysogeny Broth (LB) agar (Difco Laboratories, Detroit, MI, USA). *P. chlororaphis* strains were routinely cultured on LB or in M9 minimal salts medium supplemented with 0.2% glucose and 1mM magnesium sulphate (M9-glc) at 28 °C. Media was supplemented with the following antibiotics: ampicillin (Amp; 100 µg/ml), gentamicin (Gm; 15 µg/ml) for *E. coli*, and piperacillin (Pip; 40 µg/ml), Gm (20 µg/ml), tetracycline (Tc; 15 µg/ml) for PA23. All antibiotics were obtained from Research Products International Corp. (Mt. Prospect, IL, USA).

**Table 1:** Bacterial strains and plasmids used in the study.

**Strains, plasmids** & **primers**	**Relevant genotype, phenotype or sequence**	**Reference or source**
**Strains**		
*P. chlororaphis* PA23	PRN+PHZ+Rif^R^; wild-type (soybean root tip isolate)	Savchuk & Fernando (2004)
PA23-8	PRN^−^Rif^R^*prnBC* deletion mutant	Selin et al. (2010)
PA23-63	PHZ^−^Rif^R^*phzE*::Tn5-OT182 genomic fusion	Selin et al. (2010)
PA23-63-1	PRN^−^PHZ^−^Rif^R^*phzE*::Tn5-OT182 genomic fusion; *prnBC* deletion mutant	Selin et al. (2010)
PA23*hcn*	PA23 with the pKNOCK-Tc vector inserted into the *hcn* gene	Nandi et al. (2015)
PA23-6863	PA23 carrying pME6863; AHL deficient	Selin, Fernando & De Kievit (2012)
PA23*phzR*	PA23 with Gm^R^ marker inserted into *phzR* gene	Selin, Fernando & De Kievit (2012)
PA23*rpoS*	PA23 with pKNOCK-Tc vector inserted into *rpoS* gene	Selin, Fernando & De Kievit (2012)
PA23*gacA*	Gm^R^ marker inserted into the *gacA* gene	Selin et al. (2014)
PA23-314	Rif^R^*gacS*::Tn-OT182 genomic fusion	Poritsanos et al. (2006)
PA23-*gfp*	PA23 containing GFP expressed from pTDK-GFP	This study
PA23-8-*gfp*	PA23-8 containing GFP expressed from pTDK-GFP	This study
PA23-63-*gfp*	PA23-63 containing GFP expressed from pTDK-GFP	This study
PA23-63-1-*gfp*	PA23-63-1 containing GFP expressed from pTDK-GFP	This study
PA23*hcn*-*gfp*	PA23*hcn* containing GFP expressed from pTDK-GFP	This study
PA23-6863-*gfp*	PA23-6863 containing GFP expressed from pTDK-GFP	This study
PA23*phzR*-*gfp*	PA23*phzR* containing GFP expressed from pTDK-GFP	This study
PA23*rpoS*-*gfp*	PA23*rpoS* containing GFP expressed from pTDK-GFP	This study
PA23*gacS*-*gfp*	PA23*gacS* containing GFP expressed from pTDK-GFP	This study
*Chromobacterium violaceum* CVO26	Autoinducer synthase (*cviI*) mutant from *C. violaceum* ATCC 31532 autoinducer biosensor	Latifi et al. (1995)
**Plasmids**		
pME6863	pME6000 carrying the *aiiA* gene from *Bacillus* sp.A24 under the constitutive P_lac_ promoter	Reimmann et al. (2002)
pTdK-GFP	GFPmut3.1 gene under control of the lac promoter, contains an origin of replication for both *P. aeruginosa* and *E. coli*, Amp^R^	De Kievit et al. (2001)
pLP170	*lacZ* transcriptional fusion vector	Preston et al. (1997)
pPRNA-*lacZ*	*prnA* promoter in pLP170	Selin et al. (2010)
pPHZA-*lacZ*	*phzA* promoter in pLP170	Selin et al. (2010)
pPHZI-*lacZ*	*phzI* promoter in pLP170	Selin, Fernando & De Kievit (2012)
pPHZR-*lacZ*	*phzR* promoter in pLP170	Selin, Fernando & De Kievit (2012)
pRPOS-*lacZ*	*rpoS* promoter in pLP170	Poritsanos et al. (2006)
pGACS-*lacZ*	*gacS* promoter in pLP170	Nandi et al. (2015)

**Notes.**

Rif rifampicin Tc tetracycline Gm gentamicin Amp ampicillin

### *Acanthamoeba* strain and culture conditions

*Acanthamoeba castellanii* (ATCC 30234) was grown axenically without shaking in 20 ml of PYG medium (proteose peptone 10 g/L, yeast extract 5 g/L, glucose 10 g/L and the additives: 4 mM MgSO_4_•7H_2_O, 0.4 mM CaCl_2_, 0.05 mM Fe(NH_4_)2(SO_4_)2•6H_2_O, 2.5 mM Na_2_HPO_4_•7H_2_O, 2.5 mM KH_2_PO_4_, 0.1 M glucose and 3.4 mM sodium citrate•2H_2_O) in T75 tissue culture flasks (Sarstedt, Saint-Leonard, QC, Canada) in a humidified incubator at 25 °C. Before the experiment, cultures were washed three times with Ac buffer (PYG medium lacking proteose peptone, yeast extract and glucose) (Moffat & Tompkins, 1992) to remove non-adherent cells or any existed cysts. Amoeba trophozoite density was measured using a Neubauer cell counting chamber. To obtain amoeba cell-free supernatant, cells were grown in Ac buffer for three days at 25 °C and then filtered using 0.22 µm filters (Sarstedt). Supernatants were stored at −80 °C.

### *P. chlororaphis* PA23—Ac co-culture assays

To study amoeba-bacterial interactions, Ac trophozoites were washed three times with Ac buffer. Amoebae were adjusted to 10^6^ cells/ml, and 1-ml aliquots were transferred into wells of a 24-well plate and incubated at 28 °C for 1 h to allow adherence. Wells were washed three times with Ac buffer to remove non-adherent amoebae. Next, bacterial suspensions grown in M9-glc were washed twice with M9-glc and adjusted to 10^8^ CFU/ml. A 1-ml volume of bacteria was added to each well and allowed to incubate at 28 °C for 15 days. Growth and viability of amoeba trophozoites was determined by microscopic visualization of eosin-stained cells (dead cells stain red; live cells remain unstained; cysts are morphologically distinct) following the method of Neilson, Ivey & Bulmer (1978). Briefly, a 10-µl volume of a 0.5% (v/v) basic eosin solution was added to a 10-µl volume of Ac trophozoites grown in the presence and absence of bacteria. Ac cells were examined and enumerated with a Neubauer cell counting chamber. The number of extracellular bacteria residing in the co-culture samples was determined through viable plate counting. Amoebae grown in the absence of bacteria were used as a negative control. Experiments were repeated three times.

### Effect of secondary metabolites on Ac trophozoite viability

Cultures of PA23 strains were incubated for 3 days at 28 °C, and then cells were pelleted, and the supernatant passed through a 0.2-µm filter to remove all bacterial cells. The experiment setup was the same as described above, except that bacterial supernatant was added to the wells. The morphological changes of amoeba were monitored using an inverted microscope (Zeiss Observer Z1 inverted microscope; Carl Zeiss Microscopy GmbH, Göttingen, Germany). To determine the effect of purified compounds on Ac, amoebae were adjusted to 10^6^ cells/ml in Ac buffer containing commercially purified PRN (Sigma, St. Louis, MO, USA) at the following concentrations: 0 µg/ml (control), 0.1, 0.5, 1, 5 and 10 µg/ml, or KCN (Sigma) at the following concentrations: 0 µg/ml (control), 50, 100, 200, 400 and 800 µg/ml. For PHZ analysis, a 15-ml overnight culture of PA23-PRN^−^ grown in LB medium was used to extract PHZ following the method of Selin et al. (2010). In brief, PHZ extractions were quantified with UV-visible spectroscopy (Biochrom Ltd. Cambridge, England), and the absorption maxima for PCA and 2-OH-PHZ were measured at 367 and 490 nm, respectively. Amoeba cells were incubated with extracted PHZ at the following concentrations: 0, 10, 20, 35, and 50 µg/ml. Ac subcultures containing various concentrations of PRN, PHZ and KCN were incubated in 24-well plates at 28 °C, and amoeba viability was monitored at 1, 6, 12, 18, 24, 48 and 72 h. Five replicates were included per assay, and the experiment was repeated three times.

### Chemotaxis assays

Bacteria were grown in M9-glc medium at 28 °C for 24 h. Axenically grown Ac cultures were prepared as described above. Petri dishes (60 × 15 mm) containing 5 ml of 1.5% water agar had three wells created 20 mm apart. An aliquot of 50 µl of Ac (10^6^ cells/ml) was transferred into the centre well. One of the two outside wells contained 50 µl of “test” bacterial inoculum, while the second well contained a 50 µl suspension of PA23 WT, the *gacS* mutant, or saline as the “control” sample. A hand-crafted grid coverslip was placed underneath the plate for counting amoebae that had migrated from the centre well towards the outside wells moving under the agar. The chemotactic index was calculated based on the formula: number of amoebae migrating towards the test sample/number of amoebae moving towards the control sample. Experiments wherein the test and control wells contained the same sample, namely saline and PA23 WT were used to demonstrate equal attraction as a means of calibrating the test. Experiments were repeated three times.

### Analysis of transcriptional fusions in the presence and absence of Ac

The activity of *prnA-, phzA-, phzI-, phzR-, rpoS-,* and *gacS-lacZ* transcriptional fusions was determined in PA23 cultured in the presence and absence of Ac trophozoites. Active amoebae were adjusted to 10^6^ cells/ml in Ac buffer, as described above. Overnight bacterial cultures grown in M9-glc were adjusted to an optical density of 0.1 (2 × 10^8^ CFU/ml) prior to co-culture with Ac or amoeba-free supernatant. Samples were grown for 24, 48, and 72 h in M9-glc at 28 °C. The effect of Ac cells or cell-free supernatant on PA23 gene activity was determined by *β*-galactosidase assays (Miller, 1972) and experiments were repeated three times.

### Antifungal activity

To assess the ability of PA23 and derivative strains to inhibit the growth of *S. sclerotiorum* in vitro, radial diffusion assays were performed as described by Poritsanos et al. (2006). Bacteria were grown in M9-glc in the presence and absence of trophozoites for 72 h at 28 °C. Five replicates were analyzed for each strain and experiments were repeated three times.

### Autoinducer detection assay

AHL analysis was conducted by spotting a 5-µL aliquot of cultures onto LB agar seeded with *C. violaceum* CV026. Strain CV026 is able to detect exogenous AHLs with carbon chain lengths ranging from C4-C8, resulting in a deep purple halo surrounding the bacterial colony (Latifi et al., 1995). The diameter of purple pigment surrounding the bacterial colonies was measured (Poritsanos et al., 2006). Five replicates were analysed for each strain, and the experiment was repeated three times.

### Protease analysis

Extracellular protease production was determined qualitatively by inoculating a 5-µL volume of bacterial culture onto a 1.5% agar plate containing 2% skim milk (Difco). Protease activity was indicated by a zone of lysis surrounding the colony after 36–48 h growth at 28 °C (Poritsanos et al., 2006). Zones of clearing were measured for each strain. Data represent the average of five replicates, and the experiment was repeated three times.

### Motility analysis

To assess the impact of amoebae on PA23 flagellar (swimming) motility, bacteria were grown overnight in M9-glc in the presence and absence of trophozoites. A 5-µl volume of culture containing bacteria or bacteria plus Ac were inoculated below the surface of a 0.3% LB agar plate (Poritsanos et al., 2006). The plates were incubated at 28 °C and the swim zone diameter was measured at 24, 48 and 72 h. For the assays, five replicates were analyzed, and the experiment was repeated three times.

### Quantitative analysis of PRN and PHZ

Production of PRN was quantified by HPLC as described by Selin et al. (2010) with the following modifications. PA23 cultures started at an initial concentration of 10^8^ CFU/ml were grown in the presence and absence of the amoeba (10^6^ cells/ml) at 28 °C in 30 ml M9-glc. PRN was extracted and quantified after 96 h. Toluene was added to the culture supernatants as an internal control. Peaks corresponding to the toluene and PRN were analyzed by UV absorption at 225 nm using a Varian 335 diode array detector. For analysis of PHZ production, PA23 cultures (10^8^ CFU/ml), were grown in the presence and absence of Ac (10^6^ cells/ml) in 30 ml M9-glc at 28 °C for 72 h. PHZ was quantified according to the method of Selin et al. (2010) as described above. PRN and PHZ analysis was performed in triplicate and experiments were repeated twice.

### Statistical analysis

An unpaired Student’s t test was used for statistical analysis of PRN, PHZ, AHL production, swimming motility, AF activity and protease production. The Tukey test was applied to determine the chemotactic preference of amoebae for each of the bacterial strains. The two-way ANOVA test was applied for amoeba-bacterial co-culture assays and gene expression analysis.

## Results

### PA23 affects Ac trophozoite viability

**Figure 1:**
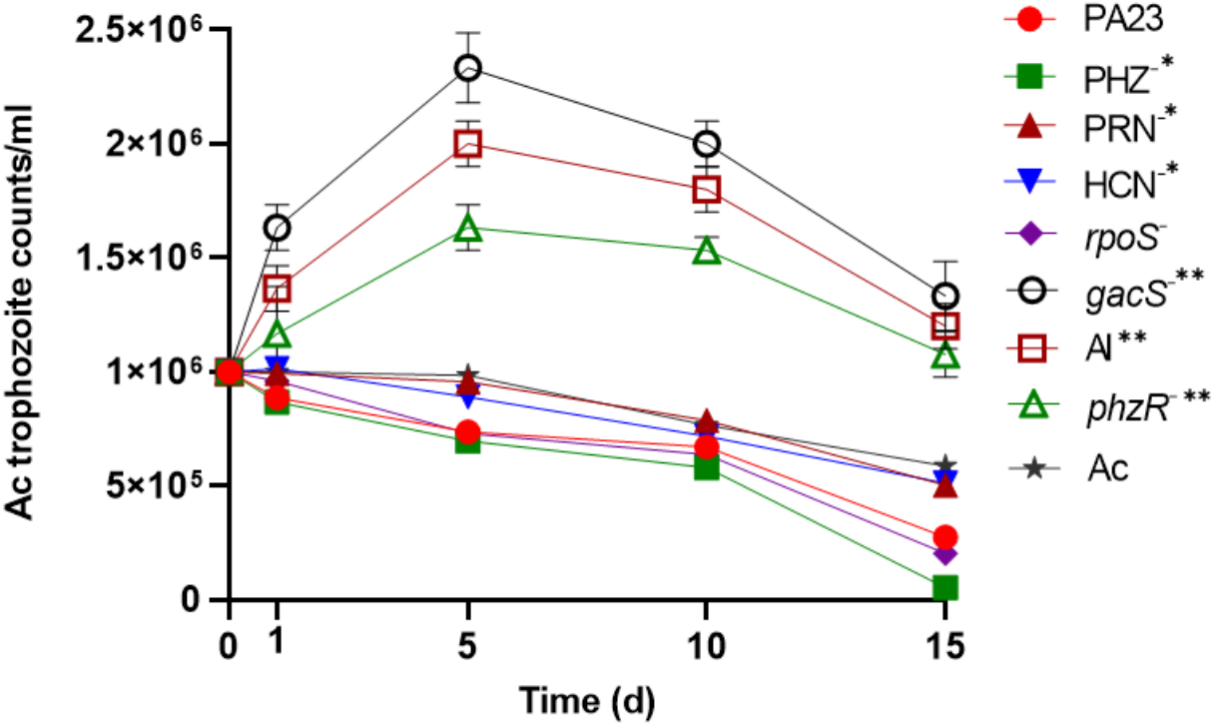
Growth of *Acanthamoeba.* trophozoites on PA23 and derivative strains in M9-glc. Amoeba growth and viability were monitored for 15 days. Asterisks indicate significant difference from the PA23 WT as determined by two-way ANOVA (*, *P* < 0.001; **, *P* < 0.0001). Note: PHZ^−^, PRN^−^ and HCN^−^ mutants are statistically significant at day 15 only, whereas the *gacS*^−^, AI-deficient, and *phzR*^−^ strains are statistically significant at days 1, 5, 10 and 15. Experiments were performed three times; one representative data set is shown.

To determine the impact of PA23 on Ac trophozoite viability and cyst formation, the PA23 WT and derivative strains, including regulatory (*rpoS*^−^*, gacS*^−^*, phzR*^−^ and AI-deficient) and biosynthetic mutants (PRN^−^, PHZ^−^, and HCN^−^) were offered as prey. Previous phenotypic analysis showed that the *gacS*^−^*, phzR*^−^ and AI-deficient strains produce little to no antibiotics and degradative enzymes (Poritsanos et al., 2006; Selin, Fernando & De Kievit, 2012). As illustrated in Fig. 1, in the presence of the *gacS*^−^*, phzR*^−^, and AI-deficient strains, Ac trophozoite numbers increased on days 1 and 5, after which the amoebae remained active but the population declined slowly. When co-incubated with PA23 WT, PHZ^−^, PRN^−^, HCN^−^ and *rpoS*^−^cells, the number of trophozoites steadily decreased over time (Fig. 1). Fig. S1 depicts the proportion of viable, encysted and dead Ac at each time point. At day 15, there were less trophozoites present in co-cultures with the PHZ^−^ strain, compared to PA23 WT. We have previously demonstrated that this bacterium and the *rpoS* mutant secrete increased levels of PRN relative to WT (Manuel et al., 2012; Selin et al., 2010). Conversely, trophozoite numbers were significantly elevated when grown on the PRN^−^ and HCN^−^ strains compared to those grown on the PHZ- strain at day 15 (Fig. 1). Collectively, these results indicate that PA23 exoproducts play a role in the inhibition of Ac growth.

### Bacterial persistence upon co-culturing with Ac trophozoites

**Figure 2:**
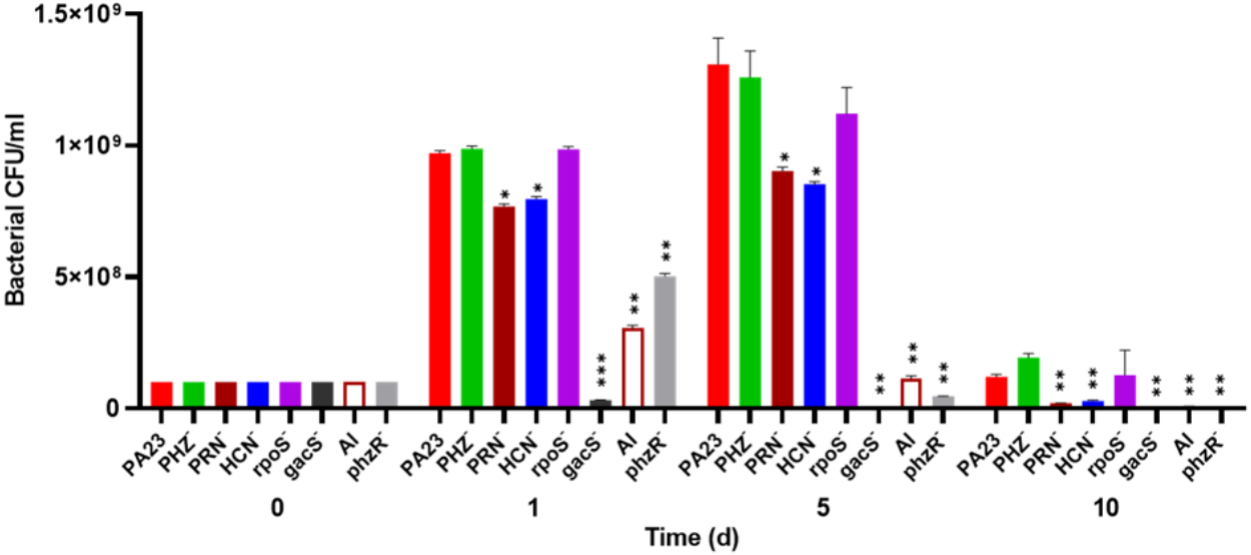
Effect of *Acanthamoeba castellani.* trophozoites on the growth of PA23 and derivative strains in M9-glc. Bacteria and amoeba were co-cultured for 15 days, and bacteria were enumerated on days 1, 5, 10 and 15. By day 15 there were no viable bacteria remaining. Asterisks indicate statistical significance of difference using two-way ANOVA (*, *P* < 0.01; **, *P* < 0.001; ***, *P* < 0.0001). Experiments were performed three times; one representative data set is shown.

To investigate whether bacterial growth was affected by the presence of amoebae, bacteria were co-cultured with Ac and viability was assessed over time. The number of PA23 WT, *rpoS*^−^ and PHZ^−^ cells increased from 10^8^ CFU/ml on day 0 to between 9.7 × 10^8^ and 9.8 × 10^8^ CFU/ml on day 1 (Fig. 2). The HCN^−^ and PRN^−^ strains also increased from 10^8^ CFU/ml to 7.5 × 10^8^ and 7.6 × 10^8^ CFU/ml, respectively. The QS-deficient *phzR*^−^ and AI^−^ derivatives showed smaller increases in population size, whereas the *gacS* numbers declined to 3.1 × 10^7^ CFU/ml. On day 5, the PA23 WT and the PRN over-producing PHZ^−^ and *rpoS*^−^ strains continued to increase in abundance. The PRN^−^ and HCN^−^ populations also increased but to a lesser degree. For the *gacS*^−^ mutant, there were no viable bacteria detected, while the number of QS-deficient cells was dramatically reduced. Bacteria populations continued to decrease by day 10, with the largest number of cells remaining for the PA23 WT, PHZ^−^ and *rpoS*^−^ strains (Fig. 2). There were no viable cells recovered on day 15 (data not shown). In the absence of Ac, there were no observable differences in bacterial viability between strains over time (Fig. S2).

### The effect of PA23 metabolites on Ac viability

**Figure 3:**
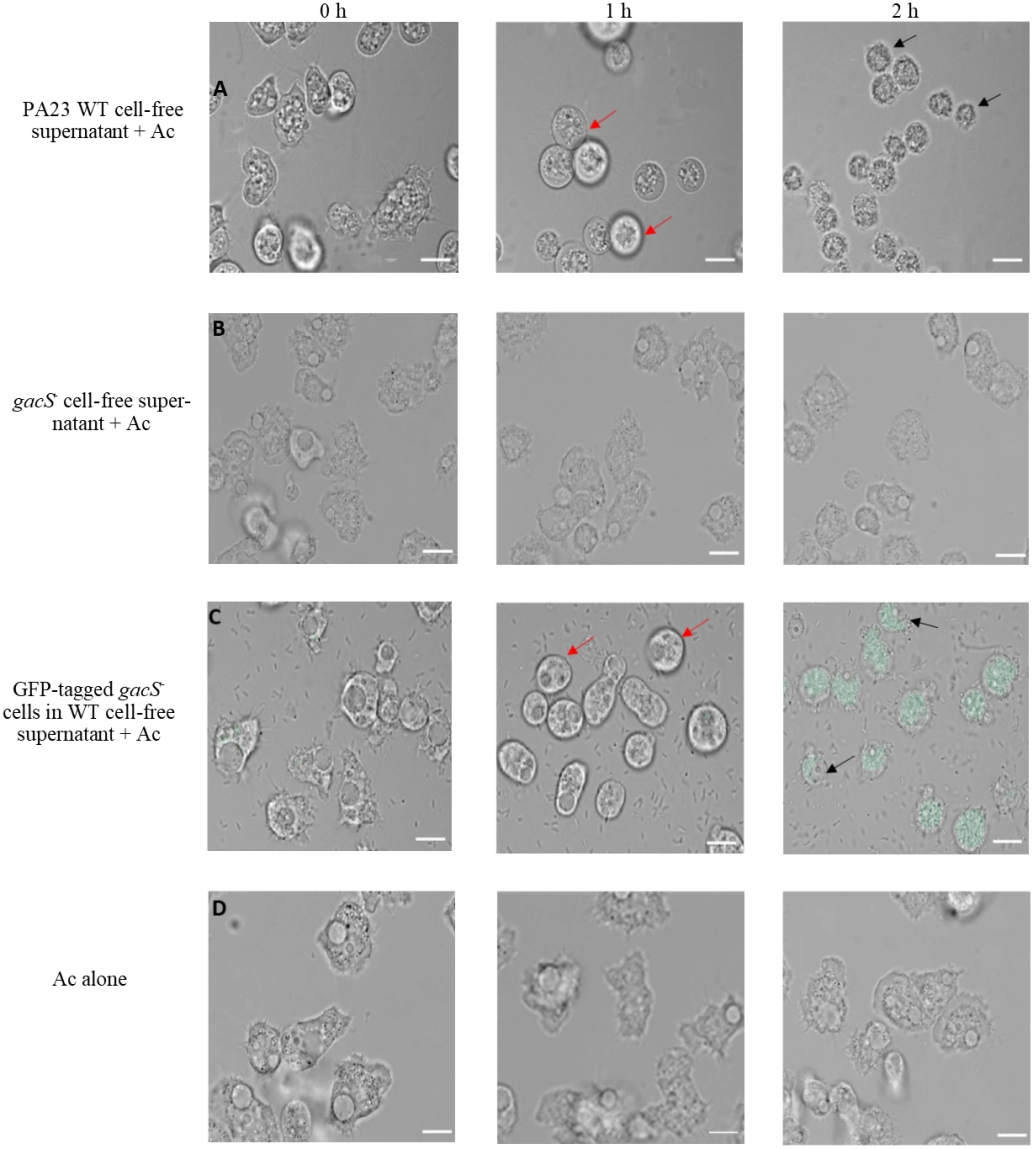
Incubation of *Acanthamoeba castellani.* trophozoites with bacterial cells and cell-free supernatant. PA23 WT cell-free supernatant (A), *gacS*-cell-free supernatant (B), GFP-tagged *gacS*-cells containing WT cell-free supernatant (C), and trophozoites in Ac buffer (D). Red arrows highlight swollen Ac trophozoites, and black arrows indicate Ac cell lysis. Images were captured using a Zeiss Observer Z1 inverted microscope under 40 × magnification. Scale bar = 10 µm.

To further explore the impact of PA23 exoproducts on Ac trophozoites, amoebae were challenged with cell-free supernatant from the PA23 WT and the *gacS* mutant (Fig. 3). After 1 h incubation with WT supernatant, amoeba cells started to swell and this continued until they began to burst at 2 h (Fig. 3A). Conversely, incubation with *gacS*^−^ supernatant did not affect amoeba morphology (Fig. 3B). Next, we investigated how feeding on nontoxic bacteria in the presence of antifungal (AF) metabolites impacts amoebae. Ac trophozoites were co-cultured with GFP-tagged *gacS*^−^ mutant cells resuspended in PA23 cell-free supernatant. As depicted in Fig. 3C, at 1 h, amoebae had lost their amoebic shape. After 2 h incubation, trophozoites were fluorescing green consistent with uptake of the *gacS*^−^ cells. Despite the fact that the trophozoites were actively feeding, they underwent the same morphological changes as when challenged with PA23 WT supernatant alone (Fig. 3A), including cell lysis (Fig. 3C). Collectively these finding indicate that secreted PA23 metabolites exert deleterious effects on Ac trophozoites.

**Figure 4:**
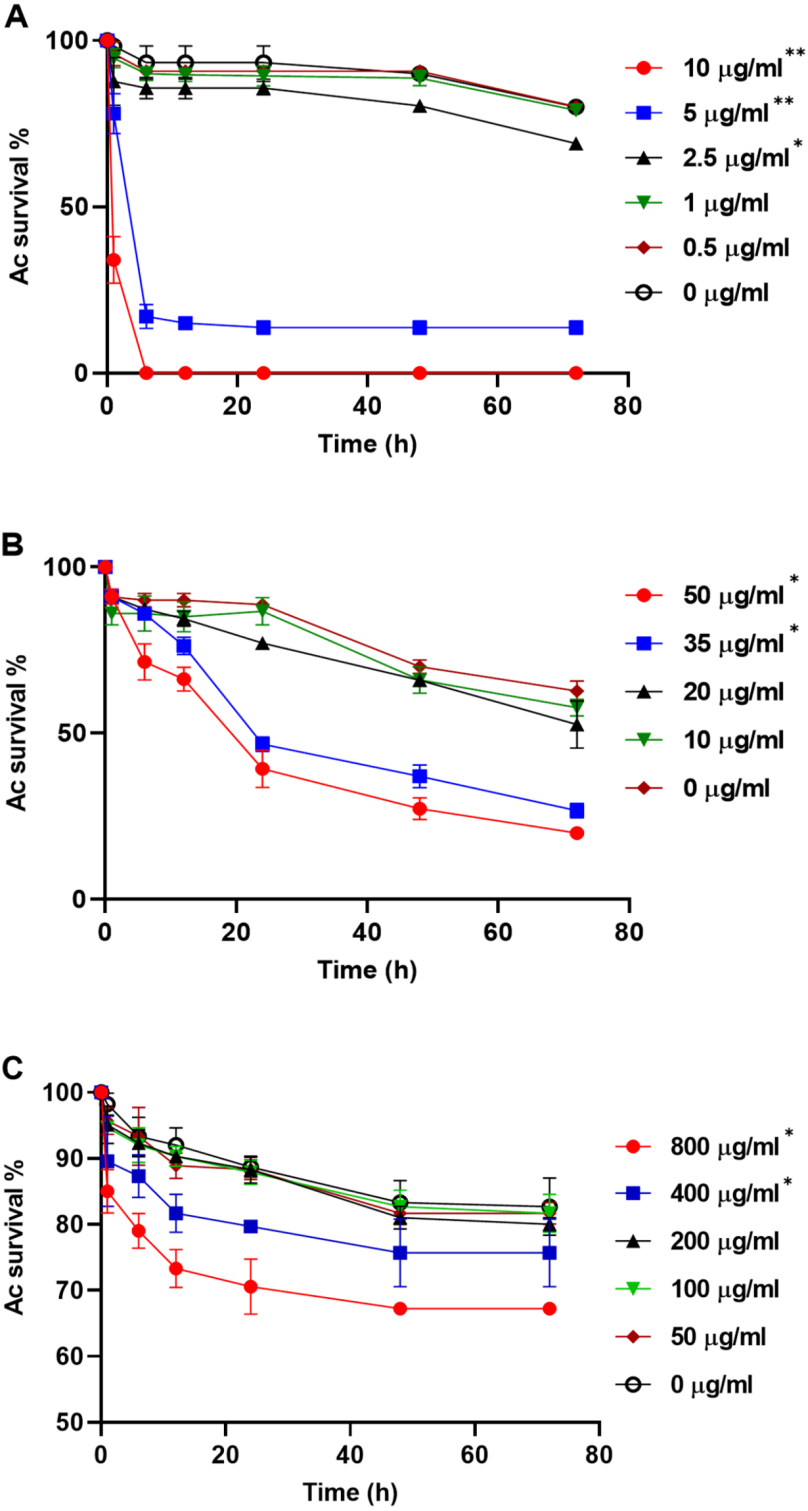
*Acanthamoeba castellani.* trophozoites challenged with PRN (0–10 µg/ml) (A), PHZ (0–50 µg/ml) (B) and KCN (0–800 µg/ml) (C**). Asterisks indicate statistical significance of difference using two-way ANOVA (*, *P* < 0.01; **, *P* < 0.001). Three replicates were used per trial, and the experiment was repeated three times. One representative data set is shown.

To explore whether purified compounds would exhibit the same toxicity, trophozoites were challenged with PRN (0-10 µg/ml), PHZ (0-50 µg/ml) and KCN (0-800 µg/ml). As illustrated in Fig. 4A, when exposed to PRN at a concentration of 1 µg/ml or lower, there was no impact on amoeba viability. However at higher PRN levels, the number of Ac trophozoites declined in a dose-dependent fashion (Fig. 4A). PHZ was also found to exhibit toxic effects on the amoebae. Exposure to ≤20 µg/ml PHZ had little effect on protozoan survival, but at higher concentrations (35-50 µg/ml), amoeba viability decreased to less than 50% after 24 h (Fig. 4B). Exposure to KCN led to a reduction in the number of Ac trophozoite at concentrations of 400 µg/ml and above (Fig. 4C).

### PA23 exoproducts affect the chemotactic response of Ac

**Figure 5:**
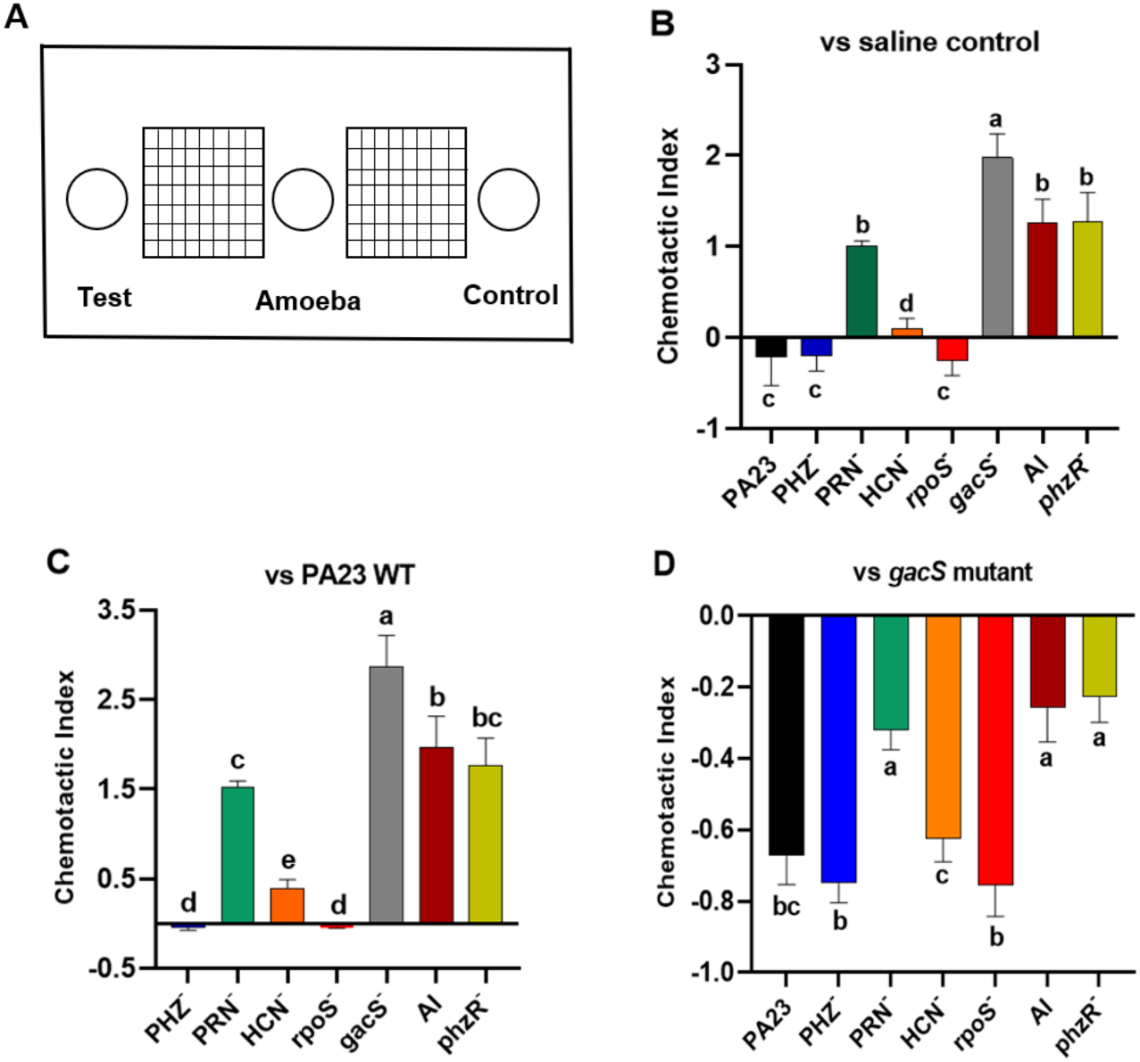
Chemotactic response of *Acanthamoeba castellani* towards PA23 WT and derivative strains. (A) Schematic diagram illustrating Petri plate set up. Active amoebae were placed in the center well; the test bacterium was placed in the test well, and PA23 WT, the *gacS* mutant or saline was added to the control well. Chemotactic preference assays were carried out against saline control (B), PA23 WT (C), and the *gacS* mutant (D). The chemotactic response was determined as follows: the number of amoebae migrating towards the test well/the number of amoebae migrating towards the control well. Values > 0 indicated attraction; values < 0 indicated repellent activity. Assays were performed in triplicate and the experiment was repeated three times. Error bars indicate ± SD; columns labelled with the same letter do not differ significantly by the Tukey test (*P* > 0.05).

Bacterial metabolites can exhibit either attractant or repellent effects, and this can ultimately impact predator grazing. To study the chemotactic response of Ac towards bacteria, binary choice assays were undertaken, as depicted in Fig. 5A. Compared to saline control, trophozoites were more attracted to the *gacS*^−^, QS-deficient, and PRN^−^ strains, all of which lack PRN production (Fig. 5B). Whereas PA23 WT, and the PRN hyper-producing PHZ^−^ and *rpoS*^−^ mutants exhibited a repellent effect. Amoebae were marginally attracted to the HCN^−^ strain (Fig. 5B). Employing PA23 as the control, amoebae preferentially migrated towards all of the strains except for the PRN overproducers (PHZ^−^ and *rpoS*^−^ mutants; Fig. 5C). Trophozoites clearly had a strong preference for the *gacS*^−^ derivative because when it was included as the control, Ac consistently migrated towards this bacterium (Fig. 5D). Once again, the PRN-producers (PHZ^−^, *rpoS*^−^, HCN^−^ and PA23 WT) exhibited the strongest repellent activity (Fig. 5D).

### Growth in the presence of Ac affects PA23 gene expression

**Figure 6:**
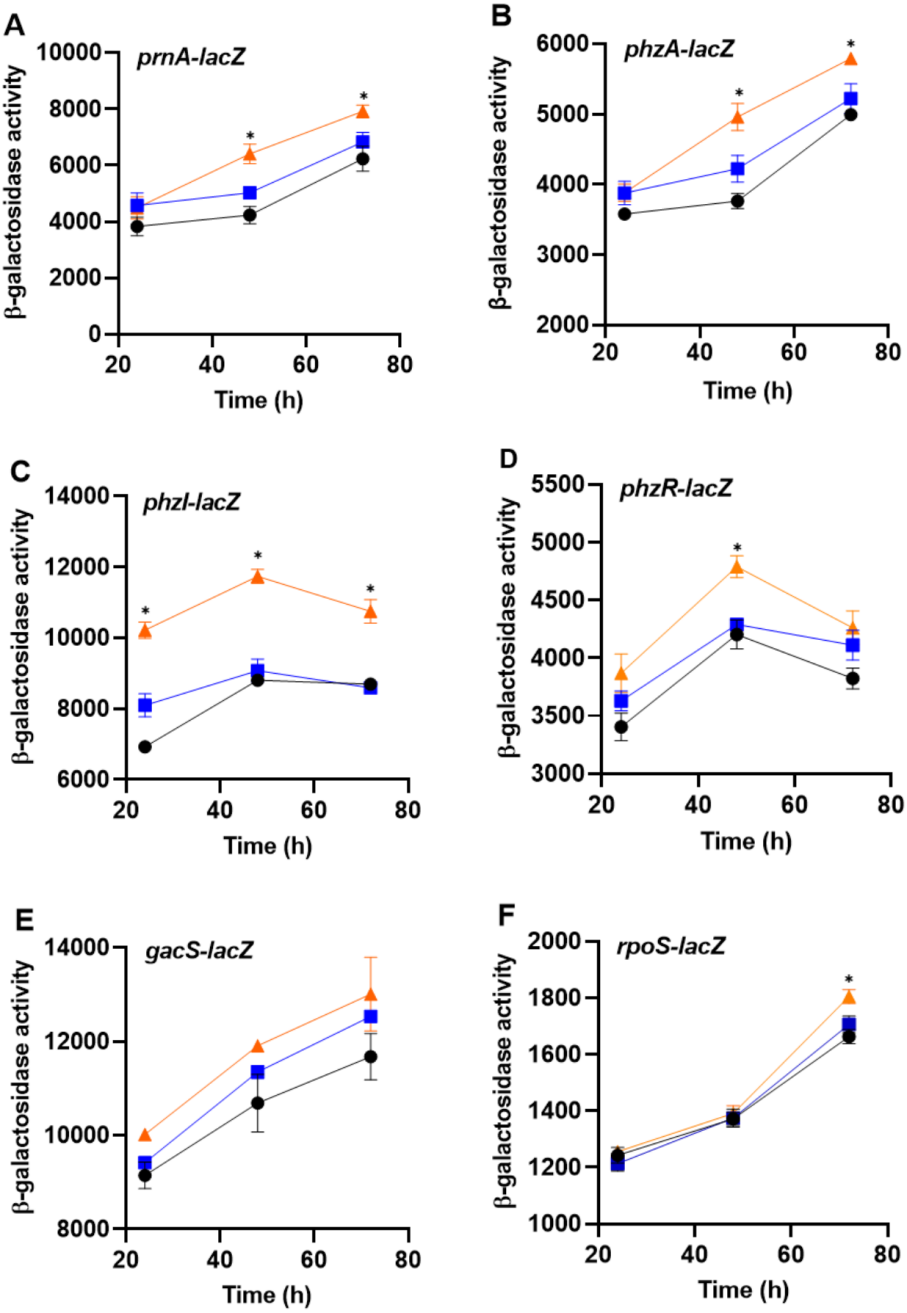
The impact of *Acanthamoeba castellani* cells and cell free supernatant on *prnA, phzA, phzI, phzR, gacS* and *rpoS* expression in *Pseudomonas chlororaphis* PA23. Co-cultures with Ac trophozoites (▴), Ac cell-free supernatant (■) and bacteria alone (•) were analyzed for *β*-galactosidase activity (Miller units) at 24, 48 and 72 h. Asterisks indicate statistical significance of difference using two-way ANOVA (*, *P* < 0.01). Experiments were performed three times; one representative data set is shown.

To determine whether bacteria can sense the presence of the predator, PA23 was grown together with amoebae cells or cell-free supernatants and monitored for changes in gene expression. For this assay, biosynthetic (*prnA* and *phzA*) and regulatory genes (*phzI*, *phzR*, *rpoS*, *gacS*) were analyzed. No changes in gene expression were observed in bacteria incubated with Ac cell-free supernatants. However, co-incubation of PA23 with trophozoites resulted in elevated expression of *phzA* and *prnA* at both 48 h and 72 h (Fig. 6). For the QS genes *phzI* and *phzR*, co-culturing resulted in a significant increase in *phzI*-*lacZ* activity at all time points tested, whereas *phzR* activity was elevated at only 48 h (Fig. 6). Growth with trophozoites led to an increase in the *rpoS-lacZ* activity at 72 h, while no change in *gacS* expression was observed at any of the time points (Fig. 6).

### Impact of Ac on PA23 phenotypic traits

Phenotypic analysis was undertaken to determine whether changes in secondary metabolite production or other traits were brought on by growth in the presence of Ac. As outlined in Table 2, co-incubation with amoebae led to increased PRN and PHZ production, consistent with the elevated *phzA* and *prnA* gene activity. Other phenotypic traits, including fungal inhibition, protease activity, and swimming motility, were unaffected by Ac (Table 2).

## Discussion

The ability of bacteria to persist in the soil is profoundly affected by grazing predators, including protozoa. In response, bacteria have developed a number of defensive mechanisms to avoid predation, such as toxin production (Jousset, 2012). The current study aimed to investigate the interaction between PA23 and the model protozoan predator Ac. Specifically, we were interested in whether PA23 AF metabolites facilitate survival in the presence of this predator and their impact on Ac viability. Additionally, we explored bacterial survival within vacuoles of trophozoites.

PA23 synthesizes an arsenal of metabolites such as PRN, PHZ, and HCN and strains deficient in these compounds exhibit altered AF activity. We have previously demonstrated that mutations in QS and the GacS-GacA two-component system abolished exoproduct formation, which in turn led to a decrease in AF activity (Poritsanos et al., 2006; Selin, Fernando & De Kievit, 2012; Selin et al., 2014). Our prey-predator co-culture assay revealed that the PA23 WT, and the PHZ^−^ and *rpoS*^−^ mutants caused a dramatic reduction in the number of Ac trophozoites either by transforming into dormant cysts or causing cell death (Fig. 1). PRN production is elevated 2.2-and 1.6-fold in PHZ^−^ and *rpoS*^−^ backgrounds, respectively (Manuel et al., 2012; Selin, Fernando & De Kievit, 2012). The increased mortality of Ac trophozoites co-cultured with these PRN hyper-producing strains led us to speculate that this antibiotic is involved in PA23 toxicity towards the predator (Fig. 1). When Ac trophozoites were challenged with different concentrations of purified PRN, viable amoebae decreased in a dose-dependent fashion (Fig. 4A). Consistent with these findings, Jousset et al. (2010) reported that purified PRN and 2,4-diacylphloroglucinol (DAPG) exhibited toxic effects towards Ac trophozoites causing rapid cell death after 6 h of incubation. In another study, the antibiotics DAPG, pyoluteorin (PLT) and PRN induced cyst formation in the amoeba *Vahlkampfia*, while the growth of amoebae was enhanced when co-cultured with toxin-deficient strains (Jousset et al., 2006). The toxicity associated with PRN is not surprising as it is known to affect a wide range of microorganisms, including fungi and protists (Chernin et al., 1996). This compound interferes with cellular processes such as respiratory pathways and osmotic regulation (Okada et al., 2005; Tripathi & Gottlieb, 1969).

**Table 2:** Phenotypic characterization of PA23 grown in the presence and absence of Ac.

**Organism**	**PHZ(µg/ml)**^a^	**PRN(µg/ml)**^a^	**Antifungal (mm)**^b^	**Protease(mm)**^b^	**AHL(mm)**^b^	**Motility(cm)**^b^
PA23 alone	32.8 (1.4)	3.4 (0.3)	5.12 (0.6)	4.87 (0.2)	4.62 (0.4)	59.6 (1.2)
PA23 + Ac	38.16 (0.9)^*^	4.4 (0.3)^*^	5.25 (0.5)	5.25 (0.5)	4.62 (0.4)	62 (0.8)

**Notes.**

a Mean ± SD obtained from five replicates.

b Mean ± SD of zones of activity obtained from five replicates.

* Significantly different from PA23 WT (*P* < 0.05).

PA23 also produces the volatile compound HCN that plays a role in AF activity (Nandi et al., 2017) and contributes to its nematicidal effects on *C. elegans* (Nandi et al., 2015). For that reason, we were interested to understand whether HCN exerts toxic effects on Ac trophozoites. We observed that Ac preferentially consumed the HCN^−^ strain and this bacterium supported slightly higher trophozoite numbers compared to PA23 WT (Figs. 1 and 2). When amoebae were incubated with purified KCN, a significant decline in the number of Ac was detected at concentrations of 400 µg/ml and higher (Fig. 4C). HCN is a broad-spectrum toxin that affects a wide range of organisms, such as fungi and nematodes (Blumer & Haas, 2000) and it also appears to inhibit Ac growth, albeit modestly.

PA23 produces two diffusible PHZ compounds, namely phenazine-1-carboxylic acid (PCA) and 2-hydroxyphenazine (2-OH-PHZ) that impart an orange colour to PA23 cells. We have previously demonstrated that PHZ production plays only a minor role in AF activity; however, it contributes to PA23 biofilm formation (Selin et al., 2010). In co-cultures, the PHZ-producing strains (WT, PRN^−^, HCN^−^, *rpoS*^−^) were less palatable than several of the PHZ-deficient bacteria (*gacS, phzR-,* AI-deficient) (Fig. 2). The one outlier being the PHZ^−^ mutant that wasn’t highly consumed, which is most likely due to the elevated levels of PRN produced by this strain (Selin et al., 2010). PHZ toxicity was further demonstrated by the fact that exposure to this compound resulted in a dose-dependent decrease in Ac viability (Fig. 4B). To the best of our knowledge, this is the first report of PHZ having amoebicidal activity. A study by Matz et al. (2004) reported that the purple pigment violacein produced by *Janthinobacterium lividum* and *Chromobacterium violaceum* is acutely toxic for the bacterivorous nanoflagellates *Bodp saltans Ochromonas* sp. and *Spumella* sp. Ingestion of WT bacteria induced rapid cell lysis whereas non-pigmented mutants supported protozoan growth. In addition, purified violacein was found to be highly toxic for the flagellates (Matz et al., 2004).

Secondary metabolites provide additional benefits if they are able to act as deterrents, allowing bacteria to avoid consumption all together. To investigate whether PA23 exoproducts exhibit repellent or attractant properties, chemotactic response assays were performed. We discovered that amoebae had a strong preference for the toxin-deficient *gacS*^−^, *phzR*^−^, and AI^−^ strains (Fig. 5). Moreover, there was very little difference between these three bacteria and the PRN^−^ strain, suggesting that PRN acts as a strong repellent (Fig. 5). We have previously shown that PRN exerts similar effects on *C. elegans* (Nandi et al., 2015). The HCN^−^ mutant, on the other hand, closely resembled PA23 WT (Fig. 3.6); therefore, HCN does not significantly impact Ac chemotaxis. Because the PHZ^−^ strain produces twice as much PRN as WT, it was not possible to assess whether PHZ affects Ac migration. While we observed only repellent effects, bacterial chemicals can also act as attractants. Gaines et al. (2019) reported that the model protozoa *Eglena gracilis* showed a positive chemotactic response towards *Listeria monocytogenes* cells. The authors suggested that the small molecules released from *L. monocytogenes* such as volatile organic compounds exhibited chemoattractant activity and were responsible for attracting *Euglena* (Gaines et al., 2019)*.* Collectively, our findings suggest that Ac trophozoites were able to sense and respond to PA23 chemical cues. Ac was only attracted to toxin-deficient strains; in particular those lacking PRN, suggesting that this antibiotic may facilitate PA23 survival in the soil.

While toxic metabolites are an effective strategy for reducing predator populations, biosynthesis of these compounds is energetically costly for the producer (Jousset, 2012). Clearly, the ability to optimize toxin production according to predation risk is beneficial for bacteria (Steiner, 2007). Therefore, we were interested to determine whether co-culturing with Ac alters expression of PA23 genes and AF products. Increased expression of *phzA* and *prnA* occurred in the presence of amoebae at 48 h and 72 h; whereas no change was observed when bacteria were incubated with Ac supernatants. Our PHZ and PRN analysis confirmed elevated production of these antibiotics (Table 2). It is interesting that the *phzI* and *phzR* QS genes were also upregulated in the presence of Ac, because the Phz QS system positively regulates *phz* and *prn* gene expression (Selin, Fernando & De Kievit, 2012). It is not clear at this time whether the effects of Ac on PHZ and PRN production are directly or indirectly mediated. We have previously shown that co-culturing PA23 with *C. elegans* led to increased *prnA* and *phzA* gene expression, while cell-free supernatants had no effect (Nandi et al., 2015). Similarly, Mazzola et al. (2009) reported that production of the cyclic lipopeptides massetolide and viscosin by *Pseudomonas protegens* SS101 and SBW25, respectively, were essential for protecting bacteria from predation by *Naegleria americana*. Moreover, the authors observed an upregulation of *massABC* (massetolide) and *viscABC* (viscosinamide) when bacteria were challenged with protozoa (Mazzola et al., 2009). In contrast to our findings, *P. protegens* CHA0 grown in the presence of Ac cell-free supernatants exhibited elevated *phlA* (DAPG) and *prnA* gene expression and increased DAPG and PRN production. However, direct contact with the predator resulted in a reduction in gene expression (Jousset et al., 2010). Collectively these findings indicate that predators and prey can sense and respond to one another, either through direct contact or soluble chemical cues.

## Conclusion

Findings presented herein demonstrate that PRN, PHZ and HCN all contribute to PA23-mediated inhibition of Ac in vitro. PA23 is able to sense the presence of amoebae and upregulate expression of genes and antipredator compounds accordingly. We have previously shown that PHZ is not essential for PA23-mediated biocontrol of the plant pathogen *S. sclerotiorum* but it is involved in biofilm formation. Intriguingly, PHZ also has amoebicidal properties. Taken together, toxins produced by PA23 exhibit broad-spectrum antagonism, not only towards fungal phytopathogens and *C. elegans,* but also Ac. Future studies on the interplay between bacteria and predators in the rhizosphere using different protists will provide additional insight into PA23 persistence in the environment.

##  Supplemental Information

10.7717/peerj.10756/supp-1 Figure S1 Bacterial survival in Ac buffer over time PA23 and derivative strains were grown in Ac buffer and cells were enumerated on day 0, 1, 5, 10 and 15. No viable cells were remaining by day 15.

10.7717/peerj.10756/supp-2 Figure S2 Total *Acanthamoeba castellani* counts showing proportion of live, dead and encysted cells in co-cultures with PA23 and derivative strains Total counts are expressed as the percentage relative to day 0, which is set at 100%.

10.7717/peerj.10756/supp-3 Data S1 Raw data for Figures and Table 2

## Additional Information and Declarations

### Data Availability

The following information was supplied regarding data availability: Raw data are available as Supplemental Files.
