# Supplementary figures and images for: Pseudomonas chlororaphis PA23 metabolites protect against protozoan grazing by the predator Acanthamoeba castellanii
Source: PeerJ. 2021 Jan 22;9:e10756. doi: 10.7717/peerj.10756 (PMC7831366; doi:10.7717/peerj.10756)

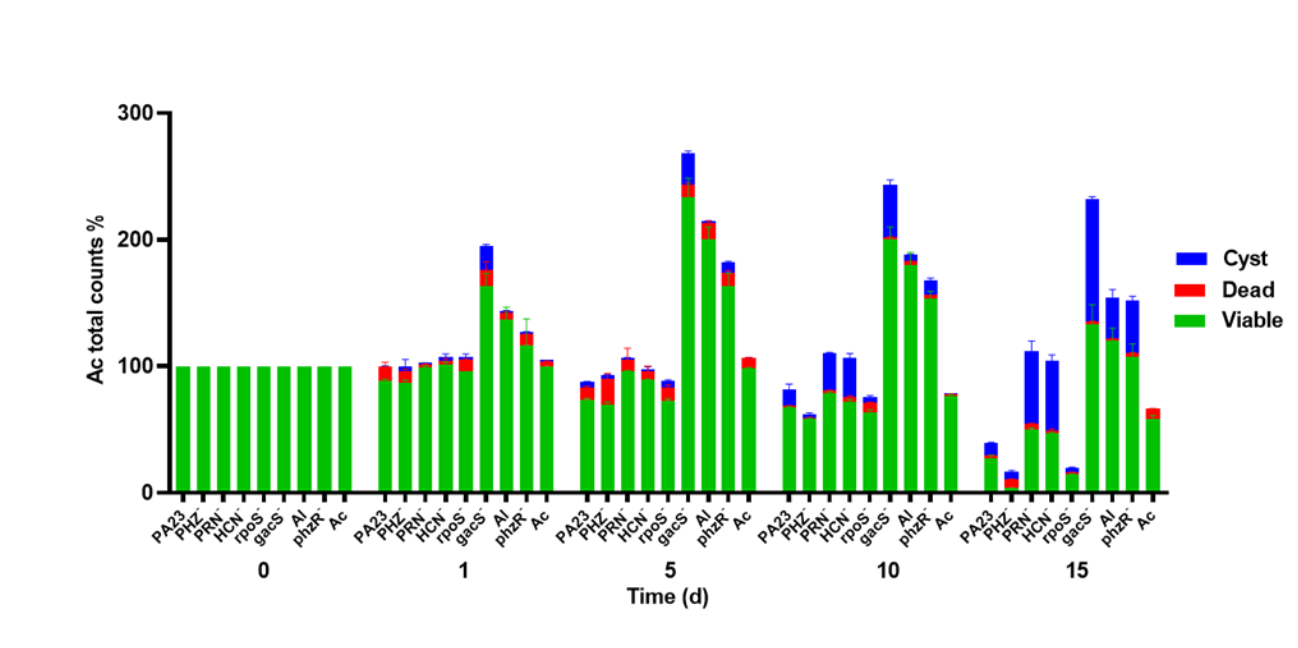

Supplement: Figure S1 — PA23 and derivative strains were grown in Ac buffer and cells were enumerated on day 0, 1, 5, 10 and 15. No viable cells were remaining by day 15. [file peerj-09-10756-s001.png]

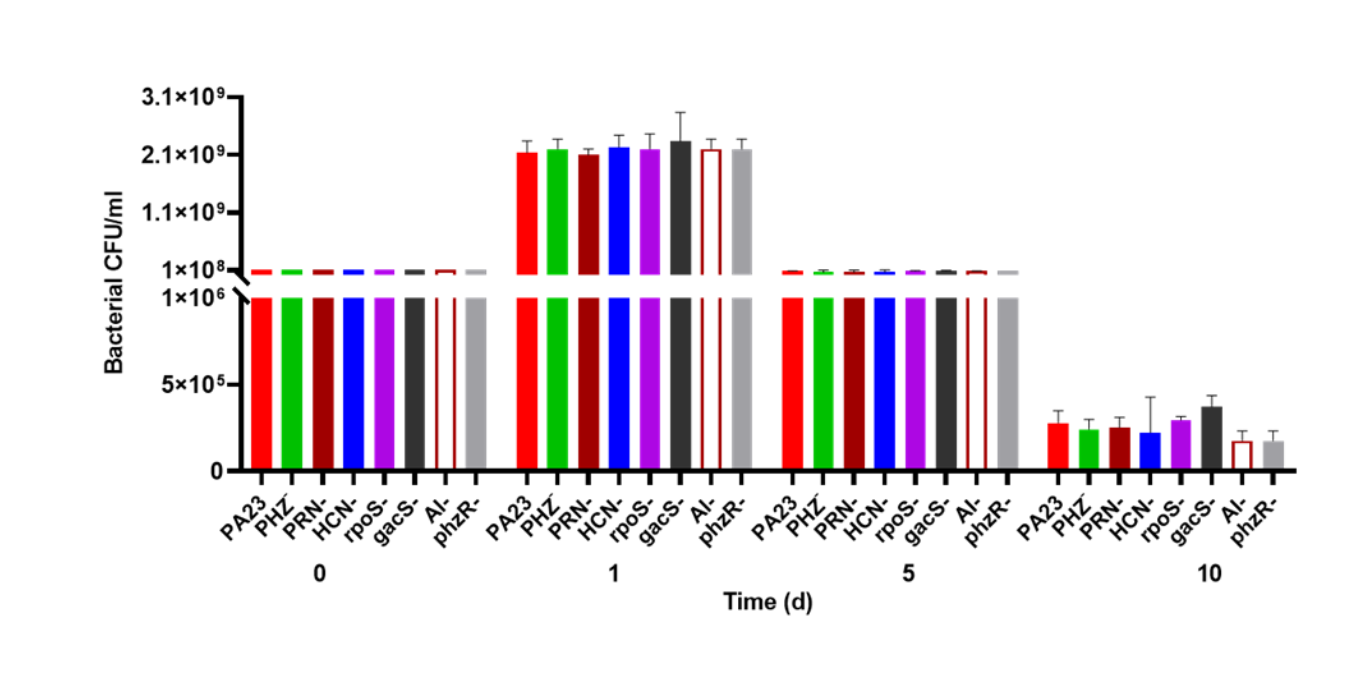

Supplement: Figure S2 — Total counts are expressed as the percentage relative to day 0, which is set at 100%. [file peerj-09-10756-s002.png]
